# The Sensitivity and Specificity of Using a Computer Aided Diagnosis Program for Automatically Scoring Chest X-Rays of Presumptive TB Patients Compared with Xpert MTB/RIF in Lusaka Zambia

**DOI:** 10.1371/journal.pone.0093757

**Published:** 2014-04-04

**Authors:** Monde Muyoyeta, Pragnya Maduskar, Maureen Moyo, Nkatya Kasese, Deborah Milimo, Rosanna Spooner, Nathan Kapata, Laurens Hogeweg, Bram van Ginneken, Helen Ayles

**Affiliations:** 1 Department of medicine, School of Medicine, University of Zambia, ZAMBART Project, Lusaka, Zambia; 2 Diagnostic Image Analysis Group, Radboud University Medical Center, Nijmegen, The Netherlands; 3 National TB program, Ministry of Health, Lusaka, Zambia; 4 Clinical research department, Faculty of Infectious and Tropical Diseases, London School of Hygiene and Tropical Medicine, London, United Kingdom; Institute of Infectious Diseases and Molecular Medicine, South Africa

## Abstract

**Objective:**

To determine the sensitivity and specificity of a Computer Aided Diagnosis (CAD) program for scoring chest x-rays (CXRs) of presumptive tuberculosis (TB) patients compared to Xpert MTB/RIF (Xpert).

**Method:**

Consecutive presumptive TB patients with a cough of any duration were offered digital CXR, and opt out HIV testing. CXRs were electronically scored as normal (CAD score ≤60) or abnormal (CAD score>60) using a CAD program. All patients regardless of CAD score were requested to submit a spot sputum sample for testing with Xpert and a spot and morning sample for testing with LED Fluorescence Microscopy-(FM).

**Results:**

Of 350 patients with evaluable data, 291 (83.1%) had an abnormal CXR score by CAD. The sensitivity, specificity, positive predictive value (PPV) and negative predictive value (NPV) of CXR compared to Xpert were 100% (95%CI 96.2–100), 23.2% (95%CI 18.2–28.9), 33.0% (95%CI 27.6–38.7) and 100% (95% 93.9–100), respectively. The area under the receiver operator curve (AUC) for CAD was 0.71 (95%CI 0.66–0.77). CXR abnormality correlated with smear grade (r = 0.30, p<0.0001) and with Xpert C_T_(r = 0.37, p<0.0001).

**Conclusions:**

To our knowledge this is the first time that a CAD program for TB has been successfully tested in a real world setting. The study shows that the CAD program had high sensitivity but low specificity and PPV. The use of CAD with digital CXR has the potential to increase the use and availability of chest radiography in screening for TB where trained human resources are scarce.

## Introduction

Recently there has been renewed interest in the role of chest x-ray (CXR) in the diagnosis of tuberculosis (TB), especially with advances that have been made with digital technology [1], [2]. Particularly, there has been an increasing interest in developing computer aided diagnostic systems for detection of TB [3]–. The advances in digital technology have made CXR cheaper, easier to use because films and chemicals are no longer needed and more reliable because automatic exposure control largely avoids unreadable images and also makes it possible to score CXRs digitally using computer aided diagnosis (CAD) [6]–[8]. In high burden TB and HIV settings, the use of CXR in TB diagnosis has been limited by the scarcity of personnel that can interpret CXR correctly but also by the large work load required to read CXRs manually [3]. Digital scoring of CXR therefore has the potential to increase the use of CXR in these settings as it does not have these limitations.

Studies have shown high variability in human scoring of CXRs with wide ranges of sensitivity and specificity [9]–[15]. Furthermore, inter-reader and intra-reader agreement also vary widely, although the use of standard classification systems for CXR scoring has improved agreement [10]–[12], [16]–[18]. On the other hand, CAD systems have the potential to reduce inter-reader and intra-reader variability and also the potential to reduce detection errors [19], [20]. Additionally, CAD is trained on digital CXR images and performance might vary with the quality of the scanned films.

The resurgence of TB has seen renewed interest and progress in new technologies for diagnosis of TB such as the molecular based tool, the Xpert MTB/RIF (Xpert) [21]–[23]. Whilst Xpert has been shown to have high sensitivity and high specificity [24]–[28], its extensive use in resource constrained settings is partly limited by its high cost [23], [29]. It has been suggested that extensive use of Xpert in these settings can be achieved by using Xpert in conjunction with other screening tests [29]. Chest radiography can be used as a pre-screening tool for TB with other more sensitive but expensive tests such as Xpert [9], [29]. In this way, the cost of diagnosis can be minimized through pre-selecting patients so that only those who are most likely to benefit from an expensive but sensitive tool have access, thus improving cost effectiveness.

As part of a TB REACH Xpert implementation evaluation project we conducted a prospective study to evaluate the use of a CAD program to digitally score CXRs of presumptive TB patients as a pre-screening test to access Xpert or Fluorescence microscopy (FM) diagnosis. In this paper, we report the sensitivity and specificity of a CAD program compared to Xpert and describe the relationship between CAD score and TB detection by Xpert, smear microscopy and clinical TB diagnosis. We also describe the association between patients presenting symptoms and bacillary load with the degree of CXR abnormality.

### Ethics statement

The study had ethical approval from the University of Zambia Ethics committee. The requirement to obtain individual written informed consent was waived for presumptive TB patients being screened as part of the case finding activities, as this was part of routine health care and all modalities of TB diagnosis included are currently recommended as best practice.

## Methods

### Study setting and study population

The study was conducted in a primary health care facility in Lusaka, Zambia. The health facility is located in peri-urban Lusaka, and offers services to a population with a high burden for TB and HIV [30]. The facility notifies over 2,000/100,000 population TB patients yearly, although a low proportion of these cases are microbiologically confirmed.

The study population comprised individuals from the catchment population of the primary health care facility that presented to the health facility and met the definition of a presumptive TB patient. A presumptive TB patient was defined as any patient of any age who presented with a cough of any duration with or without other symptoms of TB and was able to submit a sputum sample. Data presented in this study was collected between June and July 2013 from consecutive patients presenting during the study period.

### Study design and study procedures

This was a cross sectional prospective study. Enhanced case finding activities were conducted in the community with the aim of raising awareness around tuberculosis; symptoms of TB, the link between TB and HIV, and the importance of seeking care early. Patients with a cough of any duration were encouraged to visit the clinic to be assessed for TB. An open access point was established at the health facility to streamline the diagnostic process. The open access point allowed patients to present directly to the TB services without queuing up in the general outpatient clinic before being investigated for TB, thus bypassing a known barrier to TB diagnosis of busy overburdened clinics. A presumptive TB patient register was maintained at the open access point. Patients that presented to the open access point were registered by allocation of a presumptive TB patient number; socio-demographic data, and history of presenting symptoms were collected in line with requirements for clinical management of presumptive TB patients. After registration, a CXR request form and HIV counseling and testing request form with the presumptive TB patient number were printed only for patients that met the definition of a presumptive TB patient. Patients that did not meet the definition of a presumptive TB patient were directed to see a clinician for assessment and management of their symptoms. Presumptive TB patients were directed to the CXR unit (Odelca-DR, Delft Imaging Systems, The Netherlands) located within the same premises. After the CXR was done, patients were directed to the HIV counseling and testing unit where opt out HIV testing was offered in accordance with the current local guidelines for patient management.

### Procedure for automatic scoring of CXR

At the CXR unit, patients were registered by scanning in the presumptive TB patient number that had been allocated at the point of registration. The CXRs were scored by a software system (CAD4TB, version 1.08, Diagnostic Image Analysis Group, The Netherlands) [4] developed for automatic detection of abnormalities suggestive of pulmonary tuberculosis. The CAD4TB software had been trained with labeled samples to distinguish between normal and abnormal CXRs. For this purpose, 945 consecutive digital CXRs (514 abnormal, 431 normal) acquired at two sites with a high TB prevalence in Sub-Saharan Africa, located in Lusaka, Zambia, and Cape Town, South Africa, were used as the training database. The CAD4TB abnormality score was calculated by combining the output of two detection systems, a textural abnormality detection and a shape abnormality detection system. These detection systems analyze the abnormalities in the unobscured lung fields which were segmented automatically as described by van Ginneken et al. [31]. The textural abnormality system was trained with various descriptive features calculated from normal and abnormal circular patches in the image to train a *k*-nearest neighbor classifier [32] (*k-*NN) to differentiate between a normal/abnormal patch in the image. These descriptive features included moments of intensity distributions of Gaussian derivative filtered images [33] and the patch position relative to the segmented lung fields. All patches in the lung fields were classified using the trained classifier to assign a probability of being abnormal. A textural abnormality score was calculated by fusing the probabilistic labels of all the patches. The shape abnormality detection system was included in the software to handle the CXR images where the lung fields were not accurately segmented. This can occur if the CXR contains substantial parenchymal abnormalities or large amounts of pleural fluid [34]. Therefore, a shape model as mentioned in [4] was constructed using lung shapes of normal CXRs in the training dataset, which was utilized to compute a shape abnormality score between 0 and 100. A high shape abnormality score reflects a very abnormal image.

The two abnormality scores computed from the above described detection systems were used as image descriptive features to train a *k-*NN classifier to estimate the combined abnormality score for a new CXR image. The combined abnormality score is in the range of 0 to 100, where a higher score is indicative of more severe abnormalities present on the CXR image.

**Figure 1:**
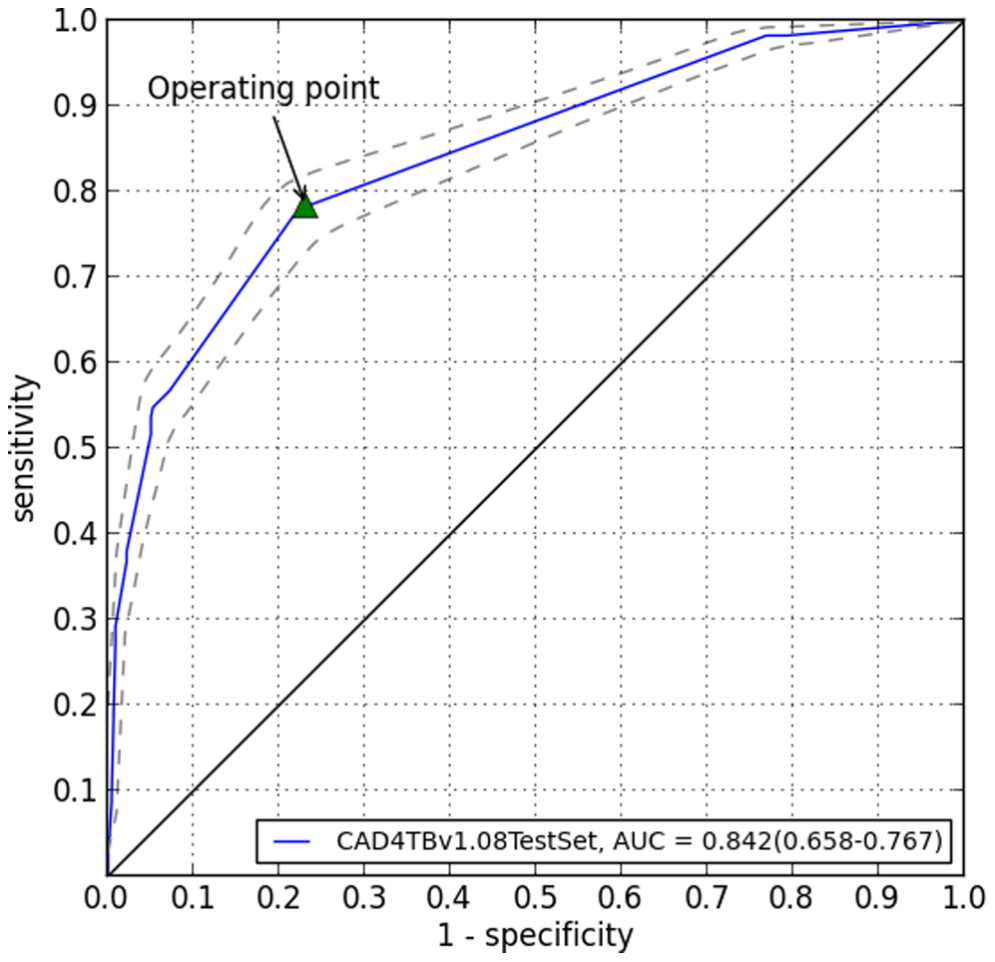
ROC on a test set of 969 CXRs.

Using previously collected CXR data from the same population, an appropriate threshold for the CAD score was determined. The area under the receiver operating (ROC) curve on a test set of 969 CXRs (458 normal, 511 abnormal) using a radiological reference standard is shown in Figure 1. A threshold of 61 was chosen based on the shown ROC curve at the operating point where 78% sensitivity and 77% specificity was achieved. For this study, a CAD score of 61 and greater was considered abnormal whilst a CAD score less than or equal to 60 was considered to be normal.

### Procedure for sputum testing and TB diagnosis

Xpert MTB/RIF (Cepheid, Inc., Sunnyvale, CA, USA) (Xpert) instruments and LED fluorescent microscopy (FM) were placed in the laboratory at the primary health care facility described above. All patients were requested to submit two spot sputum samples; one spot sample was tested with Xpert whilst the other was tested with FM. For FM testing, a further morning sample was also requested in line with the National TB Program (NTP) guidelines for testing with sputum smear microscopy. FM and Xpert testing were done according to the standard operating procedures [35], [36]. Briefly, GeneXpert lysate was added to the sputum sample using 2X the volume of the sample, the mixture was incubated for a maximum of 15 minutes with intermittent vortexing. After the incubation 2 mls of the sample were added to the Xpert cartridge and loaded into the Xpert instrument within 30 minutes. Any leftover samples were stored at 4 degrees for a maximum of 12 hours. Slides were prepared and stained using Auramine “O” stain at the primary health care facility and for blinding purposes, the slides were read by a centrally located team who were not aware of the Xpert results. Smears were graded positive for: 10 to 19 AFB were seen in 100 microscopic fields (scored as scanty positive), when 20 to 99 AFB were seen in 100 fields (scored as 1+), when 1 to 10 AFB were seen per field in at least 50 fields (scored as 2+), and when more than 10 AFB were seen per field in at 20 fields (scored as 3+). Patients that were not TB detected by either FM or Xpert underwent a clinical review of symptoms, as well as physical examination by a clinician. The clinician also reviewed the CXR independent of the CAD score to decide whether or not to commence TB treatment.

### Definition of TB diagnosis

A diagnosis of TB in this study was defined as bacteriologically confirmed TB or clinical TB. Bacteriologically confirmed TB was defined as Xpert TB detected or FM positive (AFB positive) whereas clinical TB was defined as TB treatment commenced on patients without bacteriologically confirmed TB but clinician decided to commence TB treatment based on symptoms, CXR and physical examination.

### Data Analysis

All data analysis were conducted using STATA Statistical Software (Stata Corporation Version 11. College Station, TX, USA). The performance of the CAD system was evaluated using the area under the receiver operating characteristic (ROC) curve. The ROC curve with 95% confidence interval was constructed using bootstrapping [37]. Initially a descriptive data analysis was done to describe the population that participated in the study. Patient characteristics were compared between patients with an abnormal CXR score and those with a normal CXR score. Significance testing was done using the chi-squared test and the t-test for categorical and continuous variables, respectively. The sensitivity, specificity, positive predictive value (PPV) and negative predictive value (NPV) for the CAD program were determined against Xpert TB detected. The sensitivity, specificity, PPV and NPV were initially calculated (with respective 95% confidence intervals) for the CAD program regardless of smear microscopy status. Then assuming that only patients with a smear negative result would proceed to Xpert testing, CAD program performance was also assessed restricted to patients that were smear negative. Further the sensitivity and specificity of CAD at different CAD thresholds was determined. A ROC curve comparing CAD to Xpert was constructed using Xpert TB as the reference standard. Further data analysis was performed to determine the correlation between CAD score and bacillary load using smear grade and Xpert C_T_ for microscopy and Xpert respectively. To perform this analysis, Pearson's correlation coefficient was determined with respective p-values and 95% confidence intervals. Logistic regression analysis was performed to determine the association between selected patient characteristics and symptoms with abnormal CXR. To perform this analysis, CAD score as the outcome variable was grouped to form a binary score of ≤60 and >60. In univariable analysis, statistical significance was considered to be a p value of ≤0.05 and for inclusion in the final multivariable analysis, only variables with a p value of ≤0.25 were included.

## Results

**Figure 2:**
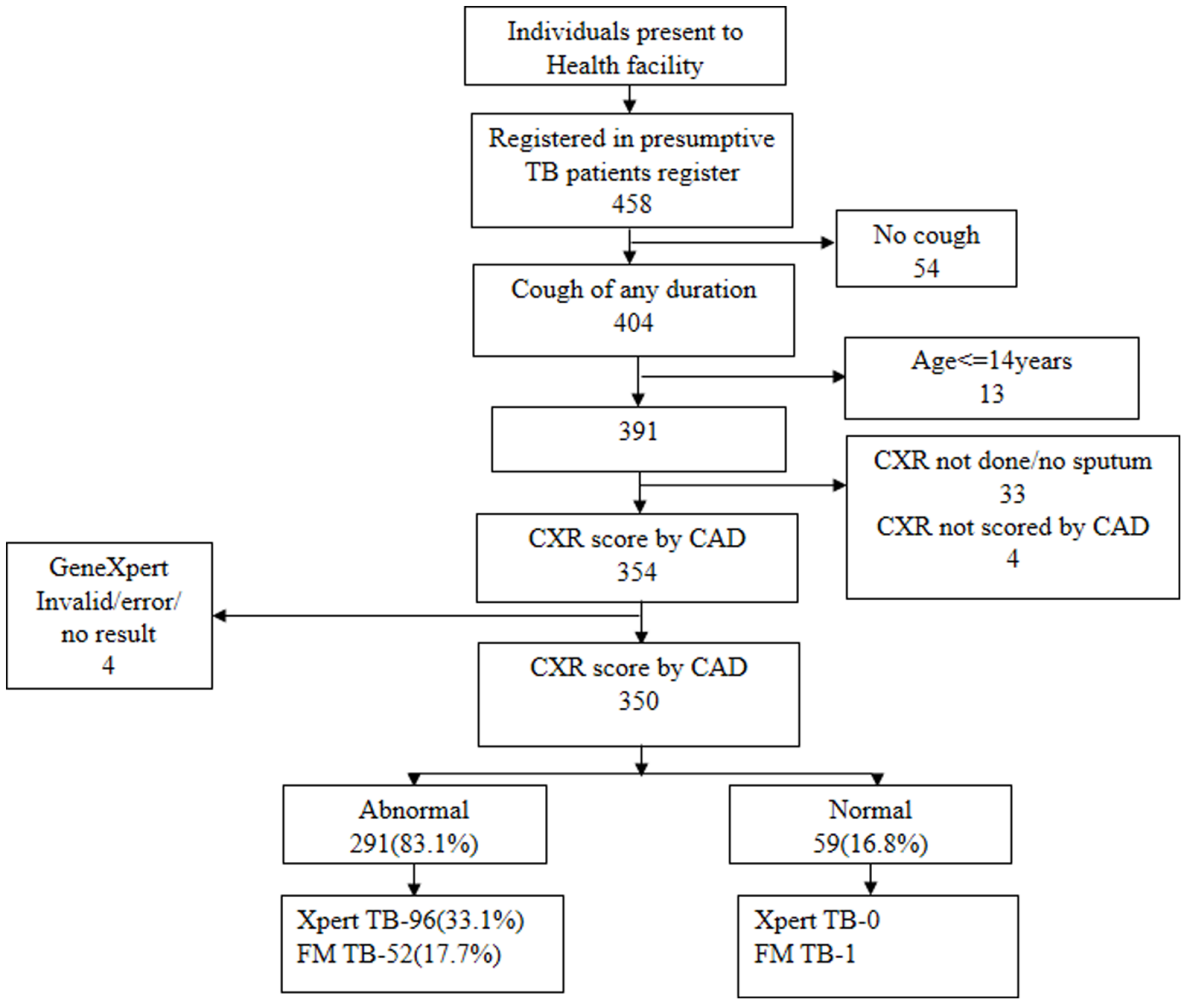
Patient flow.

Among all patients that presented to the open access point during the study period, 404/458 (88.2%) met the definition of a presumptive TB patient (figure 2). Amongst these, 13 were aged less than 15 years and were not included in the analysis. 33 patients did not have a CXR or were unable to provide a sputum sample for testing, 4 did not have a CAD reading for their CXR and 4 had an invalid Xpert result giving 350 presumptive TB patients with full data available (See figure 2). Among these 350 patients 291 (83.1%) had a CXR scored as abnormal (CAD>60) and the rest were scored as normal (CAD≤60). Among 291 patients with abnormal CAD score, Xpert detected TB in 96 (33.0%) and FM detected TB in 52 (17.9%) all of whom were also detected using Xpert. Among those with normal CXR, Xpert did not detect any TB, whilst 1 patient was FM positive (1.7%).

There were 215 (61.4%) males and 135 (38.6%) females in the study population. The mean age of study participants was 36.5 (SD 11.6). The majority of patients had a cough of duration 2–8 weeks; 226 (64.6%). HIV positive individuals made up 54.3% of the study population. The proportion of patients with an abnormal CXR was higher among HIV positive patients (p = 0.018), those with fever (p = 0.053), weight loss (p<0.0001), shortness of breath (p<0.0001) and night sweats (p = 0.025) (Table 1).

**Table 1:** Baseline characteristics of presumptive TB patients with abnormal and Normal Chest x-ray.

	All	Abnormal CXR	Normal CXR	p-Value
	350(%)	291(%)	59(%)	
**Gender**				0.007
Male	215(61.4)	187(64.3)	27(45.8)	
Female	135(38.6)	103(35.4)	32(54.2)	
**Mean age(SD)**	36.5(11.6)	36.2(11.4)	37.7(12.4)	0.369
**Cough duration**				0.912
<2week	93(26.7)	76(26.1)	17(28.8)	0.691
2–8 weeks	226(64.6)	189(64.9)	37(62.7)	0.771
>8 weeks	31(8.9)	26(8.9)	5(8.5)	0.444
**Previous TB**				0.269
No	272(77.7)	223(76.6)	49(83.0)	
Yes	78(22.3)	68(23.4)	10(16.9)	
**HIV status**				0.018
Negative	148(42.3)	115(39.5)	33(55.9)	
Positive	190(54.3)	166(57.0)	24(40.7)	
Unknown status	12(3.4)	10(3.4)	2(3.4)	
**Fever**				0.053
No	74(21.1)	56(19.2)	18(30.5)	
Yes	276(78.9)	235(80.8)	41(69.5)	
**Weight loss**				<0.0001
No	173(49.3)	131(45.0)	42(71.2)	
Yes	177(50.7)	160(55.0)	17(28.8)	
**Chest pains**				0.565
No	29(8.3)	23(7.9)	6(10.2)	
Yes	321(91.7)	268(92.1)	53(89.8)	
**Shortness of breath**				<0.0001
No	246(70.3)	192(66.0)	54(91.5)	
Yes	104(29.7)	99(34.0)	5(8.5)	
**Night sweats**				0.025
No	111(31.7)	85(29.2)	26(44.1)	
Yes	239(68.3)	206(70.8)	33(55.9)	
**Smear result**				
Negative	298(85.1)	240(82.5)	58(98.3)	<0.0001
Scanty positive	8(2.3)	8(2.7)	0	0.225
1+	12(3.4)	12(4.1)	0	0.105
2+	17(4.9)	16(5.5)	1(1.7)	0.160
3+	15(4.3)	15(5.1)	0	0.059
**Xpert Ct**				
No TB detected	254(72.6)	195(67.0)	59(100.0)	<0.0001
>28(very low TB)	16(4.6)	16(5.5)	0	0.049
22–28(low TB)	20(5.7)	20(6.9)	0	0.022
16–22(medium TB)	36(10.3)	36(12.4)	0	0.001
<16(high TB)	24(6.9)	24(8.2)	0	0.010

The sensitivity, specificity, PPV and NPV of CAD compared to Xpert were 100% (95%CI 96.2–100), 23.2% (95%CI 18.2–28.9), 33.0% (95%CI 27.6–38.7) and 100% (95% 93.9–100), respectively. The specificity of CAD among HIV positive individuals was lower than among negative individuals; 18.0% compared to 29.2% (p = 0.039). If CXRs were only done in those with a negative smear result, the performance of the CAD program did not change and was similar to when all individuals were considered (see Table 2).

**Table 2:** Sensitivity, specificity of CXR compared to GeneXpert MTB/RIF.

Compared to gold standard-Xpert TB Detected				
	Sensitivity (%)	Specificity%	PPV	NPV
	(95%CI)	(95%CI)	(95%CI)	(95%CI)
**CXR regardless of smear status N = 350**				
**All** *	96/96 (100%)	59/254 (23.2%)	96/291(33.0%)	59/59 (100%)
	(96.2–100)	(18.2–28.9)	(27.6–38.7)	(93.9–100)
**HIV positive**	57/57 (100%)	24/133 (18.0%)	57/166 (34.3%)	24/24 (100%)
	(93.7–100)	(11.9–25.6)	(27.2–42.1)	(85.7–100)
**HIV negative**	35/35 (100%)	33/113 (29.2%)	35/115 (30.4%)	33/33 (100%)
	(90.0–100)	(21.0–38.5)	(22.2–39.7)	(89.4–100)
**Smear microscopy negative followed by CXR N = 298**				
**All** *	45/45 (100%)	58/253 (22.9%)	45/240 (18.7%)	58/58 (100%)
	(92.1–100)	(17.9–28.6)	(14.0–24.3)	(93.8–100)
**HIV positive**	34/34 (100%)	24/133 (18.0%)	34/143 (23.8%)	24/24 (100%)
	(89.7–100)	(11.9–25.6)	(17.1–31.6)	(85.7–100)
**HIV negative**	10/10 (100%)	32/112 (28.6%)	10/90 (11.1%)	32/32 (100%)
	(69.1–100)	(20.4–37.9)	(5.4–19.5)	(89.1–100)

*includes Unknown HIV status.

**Figure 3:**
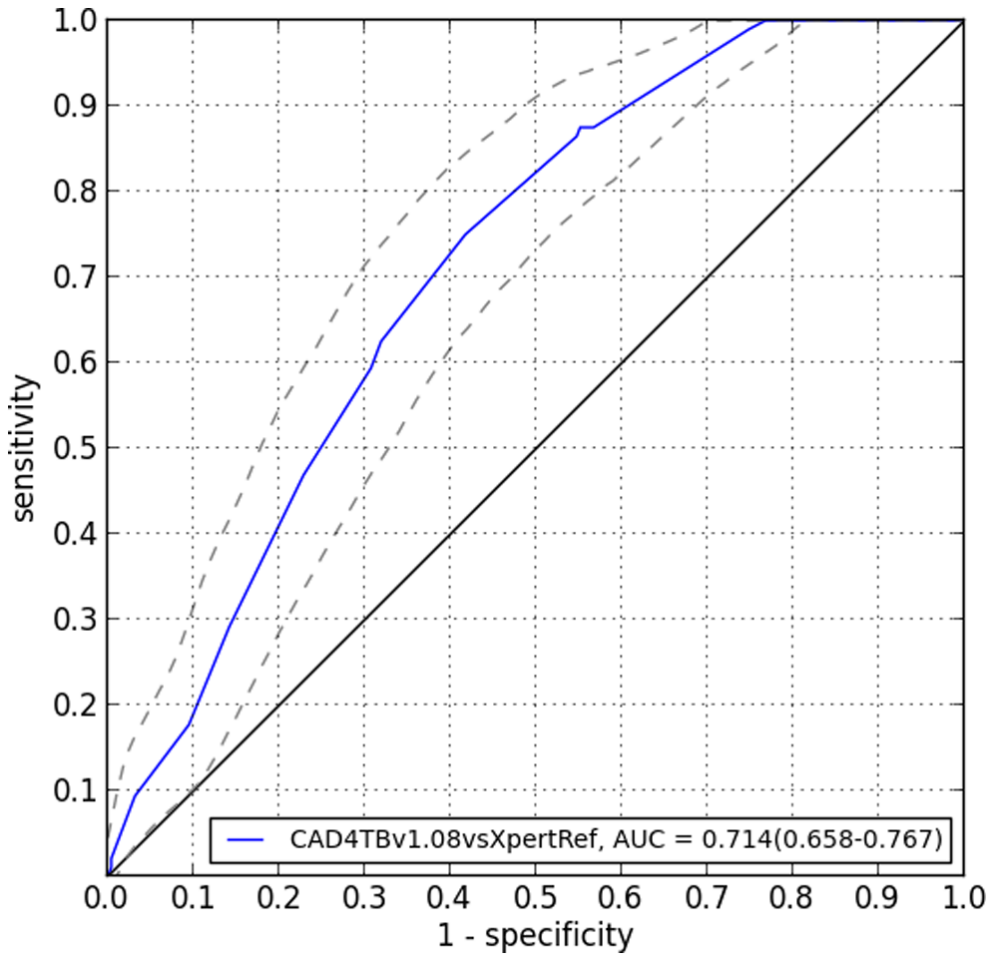
ROC curve comparing CAD score to Xpert TB as the reference standard.

Using Xpert as the reference standard, the area under the ROC curve for CAD was 0.71 (95%CI 0.66–0.77) (See figure 3). The sensitivity of CAD at different CAD threshold is shown in table 3.

**Table 3:** Sensitivity and specificity at different CAD score thresholds with Xpert TB as the reference standard.

Sensitivity	1-specificity	CAD Threshold
0	0	99
0.01	0.00	99
0.02	0.00	97
0.09	0.03	95
0.16	0.08	94
0.18	0.09	93
0.29	0.14	92
0.47	0.23	91
0.59	0.31	88
0.60	0.31	86
0.63	0.32	84
0.75	0.42	82
0.86	0.55	81
0.88	0.55	76
0.88	0.56	74
0.88	0.57	72
0.99	0.75	69
1.00	0.77	61
1.00	0.98	60
1.00	1.00	56

Comparing CAD score and bacillary load, the correlation coefficient for smear grade compared to CAD score was r = 0.30(95%CI 0.29–0.31) p<0.0001, and for Xpert C_T_, r = 0.37(95%CI 0.36–0.38) p<0.0001.

Patient characteristics associated with abnormal chest x-ray were male gender adjusted odds ratio (aOR) 2.33(95%CI 1.27–4.26); HIV positive status aOR 1.89 (95%CI 1.02–3.50); and reporting the symptom shortness of breath aOR 4.20 (1.55–11.39) (table 4)

**Table 4:** Univariable and multivariable odds ratios showing patient characteristics associated with abnormal CAD score (61–100).

	CAD score ≥61 N = 291(%)	Unadjusted OR	95%CI	Adjusted OR	95%CI
**Gender**					
Female	103 (35.4)	1.00		1.00	
Male	188 (64.6)	2.16	1.23–3.81	2.33	1.27–4.36
**Age group**					
15–24	30 (10.3)	1.00			
25–34	112 (38.5)	0.93	0.32–2.69		
≥35	145 (49.8)	0.73	0.26–2.03		
Missing	4 (1.4)	-			
**Cough duration**					
<2week	76 (26.6)	1.00	-		
2–8 weeks	189 (64.9)	1.14	0.61–2.15		
>8 weeks	26 (8.9)	1.16	0.39–3.47		
**Previous TB**					
No	223 (76.6)	1.00			
Yes	68 (24.8)	1.50	0.72–3.10		
**HIV status**					
Negative	115 (39.5)	1.00	-	1.00	
Positive	166 (57.0)	1.98	1.11–3.53	1.89	1.02–3.50
Unknown status	10				
**Fever**					
No	56 (19.2)	1.00	-		
Yes	235 (80.8)	1.84	0.98–3.44		
**Weight loss**					
No	131 (45.0)	1.00	-	1.00	
Yes	160 (55.0)	3.02	1.64–5.55	1.72	0.88–3.36
**Chest pains**					
No	23 (7.9)	1.00	-		
Yes	268 (92.1)	1.32	0.51–3.39		
**Shortness of breath**					
No	192 (66.0)	1.00	-	1.00	
Yes	99 (34.0)	5.57	2.16–14.37	4.20	1.55–11.39
**Night sweats**					
No	85 (29.2)	1.00	-		
Yes	206 (70.8)	1.91	1.08–3.39		
**Number of symptoms**					
1	3 (1.0)	1.00			
2	21 (7.2)	2.10	0.63–12.31		
3	43 (14.8)	2.87	0.52–15.77		
4	97 (33.30	5.39	1.01–28.84		
5 or more	127 (43.6)	9.77	1.79–53.42		

Overall, 97 (27.7%) patients had a confirmed diagnosis of TB by either FM or Xpert. Among patients without a confirmed diagnosis, clinical TB diagnosis was made in 71 out of 253 (28.1%) patients (results not shown).

## Discussion

To our knowledge this is the first prospective study to report the use and performance of a computer aided diagnosis system for detection of TB in a real world setting. In this high HIV prevalence setting, the CAD program performed well in both HIV positive and HIV negative patients.

The sensitivity and NPV of CAD was high, and this finding is similar to what has been shown by other studies that have assessed CXR performance for diagnosis of TB [9], [11], [29]. The difference in this study is that we assessed automatic reading of CXR whereas other works assessed CXR using human readers. Similarly, the low specificity observed in this study is similar to findings by Theron et al. who also found that specificity of CXR alone was below 30% [29] though this study assessed human scorers.

These findings demonstrate the potential role automatic CXR scoring has especially where qualified human resources are scarce. In a previous study performed in the same setting on a similar population, using the same CAD program, we observed that automatic scoring was similar to that by human readers [20]. The CAD program used in this study meets the criteria for a good screening test. Initial screening tests should have a very high sensitivity so that true positive cases will not be missed. In this study, the sensitivity of CAD compared to Xpert was 100% and expectedly the PPV was low. As shown in table 3, a higher threshold for CAD can be chosen at minimal loss of sensitivity, i.e. at a threshold of 76, 88% sensitivity and 45% specificity (much higher than reported in this study) can be achieved. This is applicable in a setting where a higher throughput is required per day, for example, where access to Xpert machines is limited.

HIV infection was significantly associated with abnormal CXR though the specificity of CAD was significantly lower in this group compared to HIV negative individuals. These findings are expected because it is known from literature that HIV positive individuals tend to present with atypical nonspecific CXR changes which can be due to a myriad number of opportunistic infections that can mimic TB in clinical presentation [38]–[41].

The AUROC for CAD of 0.71 was significantly greater than 0.5 suggesting that the ability by CAD to discriminate between patients with TB and those without TB was more than a chance occurrence. CAD seemed able to discern changes synonymous with TB to a certain degree. CAD's ability to discern changes likely due to TB are also observed through the finding that bacillary load was linearly correlated to the degree of CXR abnormality though this linear association was weak as indicated by the finding of correlation coefficients that were between the values 0.5 and −0.5.

The association between abnormal CXR and some symptoms of TB suggests that CAD performance could possibly be improved by adding symptoms. A regression model can be built that takes into account the CAD score and symptoms to estimate the likelihood of having TB and this could result in improved specificity of CAD. Different abnormal CAD score thresholds could be set depending on individual patients' clinical presentation. Validation of these findings is required with larger studies performed in various settings.

Still 56% among those with highly abnormal CXR did not have TB detected by Xpert. Patients that were Xpert or FM negative underwent clinical assessment which included review of CXR and symptoms as well as physical examination by a clinician to decide whether or not to start TB treatment. Clinical diagnosis of TB was made in 28.1% of these cases and several reasons can be advanced for this finding. Firstly, the CAD system will also score CXR changes due to pneumonia as highly abnormal. Secondly, it is well known that the sensitivity of Xpert among smear negative patients ranges from 60–80% [25], [26], [28] and in this study, only 15% of the participants were smear positive. Finally, the sensitivity of Xpert can be marginally improved when more than one sputum specimen is examined for each patient [29]. However in this study only one sample was tested per patient, and this may have limited the sensitivity of the Xpert test.

The findings in this study suggest that CAD score can be used to pre-screen patients prior to using Xpert or FM testing. Individuals with a low CAD score ≤60 are very unlikely to have TB and therefore could be offered antibiotics and follow up. In situations where resources are limited and Xpert is not available to all patients suspected of having TB then its use could be targeted by CAD score.

The major limitation of this study was that we used Xpert as the gold standard for determining the sensitivity and specificity of CAD as opposed to using mycobacterial culture. However, single Xpert test has been shown to have a sensitivity of ∼98% in smear positive samples and ∼75% in smear negative samples [28], [42]. Despite the acknowledged limitation, this study still provides useful information on the performance of CAD, especially when used in a high burden setting area where use of such technology is likely to have the most impact. Further studies are required to validate use of CAD as well as to ascertain the cost effectiveness of using CAD to prescreen patients.

## Conclusion

The advances that have been made with digital technology have made it possible to score chest radiographs automatically using computer aided diagnosis software programs. This prospective study is the first to demonstrate that CAD has the ability to discriminate CXR as normal or abnormal and that its ability to discriminate between patients with TB and those without TB was more than a chance occurrence. There is potential for roll-out of digital x-ray technology with CAD programs, especially in high burden settings where human resources are scarce. In such situations, CAD and CXR can be used as a pre-screening tool before applying more expensive diagnostic tests but cost effectiveness of such strategies would have to be ascertained. These findings need to be validated with larger studies, using mycobacterial culture as a gold standard.
